# Supplementary material for: Large variation in anti-SARS-CoV-2 antibody prevalence among essential workers in Geneva, Switzerland
Source: Nat Commun. 2021 Jun 8;12:3455. doi: 10.1038/s41467-021-23796-4 (PMC8187639; doi:10.1038/s41467-021-23796-4)
Supplement: Supplementary file 1 — Supplementary Information [file 41467_2021_23796_MOESM1_ESM.pdf]

Supplementary Information

Large variation in anti-SARS-CoV-2 antibody prevalence among essential workers in Geneva, Switzerland

Supplementary note 1: Selection and recruitment of facilities and participants

Selection of facilities and participants

As there was no list of mobilized workers during the pandemic in the canton of Geneva from which we could select participants, we selected facilities that were potentially mobilized. Public and private companies and institutions based in the canton of Geneva and that remained mostly operational with on-site activity during the lockdown were invited to apply for participation in the SEROCoV-WORK+ study, either directly or via intermediates, notably the Geneva Chamber of Commerce, Industry and Services.

This selection process was conducted as follows:

1. The SEROCoV-WORK+ Scientific Board evaluated and prepared a list of companies and institutions, and submitted it to the SEROCoV-WORK+ Integrity Board.

2. The SEROCoV-WORK+ Integrity Board, comprised of representatives from the Geneva cantonal authorities, the Geneva University Hospitals medical directorate, and the University of Geneva, made a final decision on eligible facilities.

Recruitment of participants

The human resources, medical or direction departments at each participating facility were responsible for communicating about the study and inviting their employees, using a template provided by the SEROCoV-WORK+ team. All invited employees were informed that participation in the study was fully on a voluntary basis and that their consent or refusal to participate had no effect on their relationship with their employer.

Employees who were in quarantine or isolation, or experiencing COVID-19-related symptoms during the study recruitment period were ineligible for participation.

Study participation

Three testing centers were selected for the SEROCoV-WORK+ study, distantly located across the main urban area of the Geneva canton to allow most employees to find one study testing center at a reasonable distance from their home or work address. Participants came to one of these three testing centers of the SEROCoV-WORK+ study for an interview and blood collection appointment that lasted 20 minutes. Participants signed a free and informed consent form, and completed a questionnaire with questions related to their employment, work-related activities and preventive measures during the spring 2020 lockdown, smoking behavior, and history of comorbidities and COVID-19-related symptoms. Participants also provided a 5-mL sample of venous blood to be tested for anti-SARS-CoV-2 IgG antibodies. In the case of some facilities, after identification of critical constraints associated to their participation at the testing centers, the study testing team (constituted of research assistants and nurses) went itself to the premises of the facility to allow the employees to participate in the study directly at their working place. The procedures followed in these cases were the same as those at the testing centers. We found no indication of testing site-induced bias after data collection (see Supplementary table 7).

Supplementary table 1. Description of activity sectors

Sector	Number of participating facilities	Description
Healthcare	10	Employees working in hospitals, clinics or ambulance companies, midwives.
Public administration	13	Cantonal and communal government administration employees
Transportation	7	Employees of public/private persons/goods transportation companies
Nursing homes	24	Nursing home employees
Public security	13	Police officers, civil protection, firefighters
Food industry	6	Employees of supermarkets, food markets
Homecare	3	Homecare nurses/assistant nurses, caregivers, personal care, companions, housekeepers
Social work	9	Employees of social aid, social assistance, social work
Financial services	5	Employees of banks
International organizations	3	Employees of various international organizations
Early childhood education	9	Employees of early childhood education centers/crèche
Pharmacy	18	Employees in pharmacies
Construction	4	Employees of construction companies
Media	2	Employees of media companies
Agriculture	6	Employees of agricultural businesses
Other businesses	18	Employees of other public or private businesses

Supplementary table 2. Description of occupations

Occupation	N	Description of jobs
Doctor/surgeon	371	Medical doctor and surgeon
Nurse/assistant nurse	1528	Nurse and assistant nurse
Other healthcare personnel	850	Other healthcare professional, biomedical/laboratory technician
Firefighter/EMT	275	Firefighter, first aid responder, EMT
Pharmacist/assistant pharmacist	206	Pharmacist, assistant pharmacist, pharmacy apprentice/intern
Domestic care worker	130	In-home caregiver, personal care assistant, companion, housekeeper
Cashier	139	Cashier /sales worker
Home delivery driver	42	Delivery driver, courier, dispatch rider
Public transport driver	349	Bus/tram driver, reduced mobility person driver, ticket controller
Taxi driver/chauffeur	36	Taxi driver, chauffeur
Gas station worker	14	Gas station worker/personnel
Police officer	696	Police officer or gendarme, civil service, army/soldier, civil protection
Bank/post office teller	100	Teller/counter agent in banks and post offices
Banking reception	99	Receptionist at banks
Cleaner	329	Cleaning, washing, and maintenance service worker
Security guard	207	Security or surveillance agent, night watch, etc.
Construction and food craft/trades	501	Construction worker, mechanic, metal worker, artisan, watch maker, electrician, electro-mechanic, warehouse worker, handler, repair person; food-processing worker, butcher, slaughterhouse worker, dairy worker, cheesemaker; hairdresser
Lorry driver	32	Truck/lorry long-haul driver
Teacher	35	Teacher, teaching assistant, tutor
Childcare worker	185	Childcare worker, childcare educator, assistant childcare educator
Farmer/gardener	104	Farmer, nursery owner, gardener, horticulturalist, winegrower, winemaker
Kitchen staff	159	Cook, assistant cook, kitchen helper, food server, etc.
Health researcher/research personnel	28	Healthcare researcher, research associate, research assistant
Social worker	578	Social worker, assistant social worker, family worker, youth worker, etc.
Journalist	179	Journalist, photographer, camera person, stage manager, etc.
Administration/HR	1610	Administrative, human resources, secretary, accounting employee
Communication/marketing manager	104	Communication or marketing manager, assistant manager
Finance, management, law, engineering	735	Engineer, data analysts, statistician, computer engineer/specialist, IT support; financial analyst, business analyst, cash management technical officer, compliance officer, accountant, consultant, fiscal controller, architect, civil engineer, investor; lawyer, court clerk, legal expert, judge, magistrate, prosecutor, air traffic controller, flight coordinator, chemist
Supermarket/food market personnel	257	Personnel working in supermarkets or small food markets/stores
Manager/assistant manager	420	Manager and assistant manager in customer service roles, apprentice/intern in customer service
Undertaker	39	Undertaker, funeral home agent, gravedigger

Supplementary methods. Statistical model for relative risk estimation

The aim of this analysis is to estimate the relative risks for SARS-CoV-2 infection in Geneva's working population using serological testing.

Following Stringhini *et al*. 2020, we start by estimating the probability that each person in the serosurvey is seropositive using a Bayesian regression model that accounts for individual characteristics and the sensitivity and specificity of the ELISA assay:

$$\begin{aligned} x_i &\sim Bernoulli\big(p_i\theta^+ + (1-\ p_i) * (1-\ \theta^-)\big) \\ logit\ (p_i) &= \mathrm{X}_i\beta \\ x^+ &\sim Binomial(n^+, \theta^+) \\ x^- &\sim Binomial(n^-, 1-\theta^-) \\ \theta^+ &\sim Uniform(0,1) \\ \theta^- &\sim Uniform(0,1) \\ \beta &\sim Normal(0,1) \end{aligned}$$

where x_i is the result of the IgG ELISA for the ith person (i = 1,…, N = 10513) in the WORK+ serosurvey. The sensitivity, θ^+, is determined using n^+ RT-PCR positive controls from the lab validation study (Meyer *et al*. 2020), of which x^+tested positive. The specificity, θ^-, is determined using n^- pre-pandemic negative controls, of which x^- tested positive.

The probability of observing a diagnostic positive is a function of the true positive rate and the false negative rate with regards to the true underlying probability of seropositivity p_i for that person. This probability itself is a function of covariates X and their coefficients β. Covariates always included sex and age categories. To estimate the relative risks of being seropositive for different groups of participants (e.g., by educational level, BMI, smoking status, etc.), we sampled from the posterior distribution of parameters and for each draw we calculated the predicted probability of being seropositive for a single group (e.g., $p_a = logit^{-1}(\beta_0 + \beta_1\mathrm{X}_1 + \beta_2\mathrm{X}_2)$ vs $p_b = logit^{-1}(\beta_0 + \beta_2\mathrm{X}_2)$) compared to its reference. We define the relative risk as the ratio of these in each draw and summarize through taking the mean and 2.5th and 97.5th quantiles of the distribution to give 95% central credible intervals.

The above model was used to estimate all of the relative risks presented except for those pertaining to the activity sectors. To estimate sector risks we use the above model but also account for facility-level clustering through random effects:

$$\begin{aligned} x_i &\sim Bernoulli\big(p_i\theta^+ + (1-\ p_i) * (1-\ \theta^-)\big) \\ logit\ (p_i) &= \alpha_c\mathrm{X}_i\beta \\ \alpha_c &\sim Normal(\mu_s, \sigma_s^2) \\ x^+ &\sim Binomial(n^+, \theta^+) \\ x^- &\sim Binomial(n^-, 1-\theta^-) \\ \theta^+ &\sim Uniform(0,1) \\ \theta^- &\sim Uniform(0,1) \\ \beta &\sim Normal(0,1) \\ \mu_s &\sim Normal(0,1) \\ \sigma_s &\sim Normal^+(0,1) \end{aligned}$$

We account for facility-level infection clustering with a random effect for facility, α_c $(c = 1, \dots, C = 150)$. We implement partial pooling for facility-level random effects by specifying sector-specific means, μ_s, and variances σ_s^2 $(s = 1, \dots, S = 16)$.

The priors on the sensitivity and specificity were flat from 0 to 1, equivalent to $Uniform(0,1)$. We used standard normal $Normal(0,1)$ priors for the logistic regression coefficients β and for the facility

random effects μ_s. The standard deviation for the facility effects was a positive half-normal, $\sigma_s \sim \mathcal{N}(0,1)$.

To estimate the relative risk for a reference individual (female, 18-34 years) in a sector s relative to one in the reference sector (healthcare), we used the following equations for each posterior draw of parameters β and σ:

$$p_s = \int_0^1 logit^{-1}(\mathrm{X}\beta + \mu_s + \sigma_s * \Phi^{-1}(t))dt$$

$$p_{ref} = \int_0^1 logit^{-1}(\mathrm{X}\beta + \mu_{ref} + \sigma_{ref} * \Phi^{-1}(t))dt$$

$$RR_s = p_s / p_{ref}$$

where $\Phi^{-1}(t)$ is the quantile function of a standard normal distribution. We estimated the seroprevalence for the population in category s (p_s) by integrating across all values of a logit-normal distribution with the standard deviation defined by the facility random effect σ_s. We then divided that quantity by the estimated seroprevalence for the reference category (p_{ref}) to calculate the relative risk (RR_s).

Models were implemented in the Stan probabilistic programming language. Every sampling run was for 1500 iterations for each of 4 chains, with 250 warmup samples discarded per chain, giving a total of 5000 posterior samples.

Alternatively, where appropriate, binomial confidence intervals for seropositivity were calculated using the Wilson method as implemented in the R function Hmisc::binconf.

Supplementary table 3. Sample description and prevalence of anti-SARS-CoV-2 IgG antibodies excluding participants fully confined during the lockdown, SEROCoV-WORK+ study, May-September 2020, Geneva, Switzerland

	N (%)	Seropositive	Relative Risk (95% CrI)
Total	9430	899 (9.5)	--
Women	5278 (56.0)	533 (10.1)	1.00 (ref.)
Men	4152 (44.0)	366 (8.8)	0.86 (0.73-0.99)
Age group, years			
18-34	2447 (25.9)	265 (10.8)	1.00 (ref.)
35-49	4038 (42.8)	408 (10.1)	0.92 (0.78-1.08)
50-65	2945 (31.2)	226 (7.6)	0.66 (0.51-0.81)
Educational level[a]			
Mandatory education	801 (8.8)	79 (9.8)	0.76 (0.49-1.10)
Apprenticeship	2024 (22.1)	158 (7.8)	0.55 (0.34-0.78)
Secondary education	1928 (21.1)	175 (9.0)	0.65 (0.44-0.92)
University	4031 (44.0)	417 (10.3)	0.76 (0.54-1.04)
Doctorate	367 (4.0)	45 (2.2)	1.00 (ref.)
Activity sector[b]			
Healthcare	1640 (17.4)	181 (11.0)	1.00 (ref.)
Transportation	993 (10.5)	81 (8.1)	0.68 (0.33-1.24)
Nursing homes	1054 (11.2)	147 (13.9)	1.21 (0.67-1.98)
Public administration	849 (9.0)	67 (7.8)	0.76 (0.30-1.52)
Public security	1024 (10.9)	78 (7.6)	0.54 (0.24-0.95)
Food industry	692 (7.3)	71 (10.2)	0.98 (0.48-1.78)
Homecare	703 (7.5)	81 (11.5)	0.95 (0.36-1.91)
Social work	666 (7.1)	54 (8.1)	0.67 (0.27-1.32)
Financial services	393 (4.2)	48 (12.2)	1.10 (0.50-2.03)
International Organizations	353 (3.7)	16 (4.5)	0.40 (0.09-1.25)
Early childhood education	230 (2.4)	12 (5.2)	0.35 (0.09-0.84)
Pharmacy	248 (2.6)	28 (11.2)	0.89 (0.38-1.63)
Construction	142 (1.5)	9 (6.3)	0.54 (0.10-1.48)
Media	129 (1.4)	5 (3.8)	0.55 (0.07-1.93)
Agriculture	89 (0.9)	8 (8.9)	0.74 (0.15-1.77)
Other	225 (2.4)	13 (5.7)	0.45 (0.13-0.98)
Out-of-work exposure[c]			
0	8195 (87.0)	684 (8.4)	1.00 (ref.)
≥1	1223 (13.0)	214 (17.5)	2.21 (1.86-2.65)
Smoking			
Non-smoker	5494 (58.3)	631 (11.4)	1.00 (ref.)
Ex-smoker	1470 (15.6)	121 (8.2)	0.69 (0.53-0.86)
Smoker	2464 (26.1)	147 (5.9)	0.43 (0.29-0.55)
BMI group			
18-24.9	5124 (55.7)	508 (9.9)	1.00 (ref.)
25-29.9	2970 (32.3)	271 (9.1)	0.97 (0.81-1.16)
≥30	1109 (12.1)	102 (9.2)	0.97 (0.74-1.23)
Chronic conditions[d]			
None	8378 (88.9)	792 (9.4)	1.00 (ref.)
1	913 (9.7)	93 (10.1)	1.18 (0.91-1.48)
≥2	138 (1.5)	14 (10.1)	1.23 (0.59-2.00)

Results are N (%), or as stated. Relative risks (95% credible interval) are from Bayesian logistic regression models, and are adjusted for test performance, age and sex. These pertain to the reference individual (Female, 18-34). 69 participants were excluded due to missing serology or sociodemographic data, or due to being outside target age range of 18-65 years. All data are self-reported except serology.

[a] Each category indicates the highest level of education attained by participant; mandatory education indicates 15 years of primary education as the highest obtained degree.

[b] See Supplementary Table 1 for details.

[c] Out-of-work exposure to confirmed COVID-19 cases.

[d] Chronic conditions: hypertension, diabetes, cardiovascular disease, respiratory disease, cancer.

Supplementary table 4. Seropositivity rate by activity sector and out-of-work exposure to confirmed COVID-19 cases, SEROCoV-WORK+ study, May-September 2020, Geneva, Switzerland

			Exposure to out-of-work confirmed COVID-19 case	
	Total sample		None	≥1
Activity sector	N	Seropositive, n (%)	Seropositive, n (%)	Seropositive, n (%)
Healthcare	1668	185 (11.1)	151 (10.1)	34 (19.4)
Transportation	1184	99 (8.4)	74 (7.0)	25 (19.7)
Nursing/care homes	1098	155 (14.1)	116 (12.6)	39 (21.9)
Public administration	1055	94 (8.9)	70 (7.7)	24 (17.1)
Public security	1053	82 (7.8)	62 (6.8)	20 (14.6)
Food industry	754	76 (10.1)	58 (8.8)	18 (18.9)
Home care services	753	91 (12.1)	61 (9.8)	30 (22.6)
Social work	734	62 (8.4)	47 (7.4)	15 (15.5)
Financial services	528	65 (12.3)	52 (11.0)	13 (23.6)
International organization	419	24 (5.7)	21 (5.8)	3 (5.6)
Other businesses	291	21 (7.2)	18 (6.9)	3 (10.3)
Early childhood education	259	15 (5.8)	8 (4.0)	7 (11.9)
Pharmacy	254	28 (11)	16 (7.9)	12 (23.1)
Construction	181	11 (6.1)	10 (6.0)	1 (7.1)
Media	166	7 (4.2)	6 (3.9)	1 (7.7)
Agriculture	97	8 (8.2)	5 (5.6)	3 (42.9)

Supplementary table 5. Seropositivity rate by occupation and out-of-work exposure to confirmed COVID-19 cases, SEROCoV-WORK+ study, May-September 2020, Geneva, Switzerland

			Exposure to out-of-work confirmed COVID-19 case	
	Total		None	≥1
Occupation	N	Seropositive, n (%)	Seropositive, n (%)	Seropositive, n (%)
Administration/HR	1608	131 (8.1)	105 (7.5)	26 (12.6)
Nurse/assistant nurse	1525	220 (14.4)	170 (13.0)	50 (22.7)
Other healthcare personnel	849	89 (10.5)	63 (8.6)	26 (22.2)
Finance, management, law, engineering	735	70 (9.5)	55 (8.4)	15 (19.5)
Police officer	696	57 (8.2)	40 (6.8)	17 (15.6)
Social worker	578	48 (8.3)	37 (7.5)	11 (12.6)
Construction and food craft/trades	501	35 (7.0)	32 (6.9)	3 (8.6)
Manager/assistant manager	420	44 (10.5)	28 (7.7)	16 (28.1)
Doctor/surgeon	371	40 (10.8)	28 (8.5)	12 (28.6)
Public transport driver	349	28 (8.0)	24 (7.3)	4 (21.1)
Cleaner	329	26 (7.9)	19 (6.6)	7 (17.5)
Firefighter/EMT	275	25 (9.1)	24 (9.7)	1 (3.6)
Supermarket/food market personnel	256	27 (10.5)	18 (8.3)	9 (23.1)
Security guard	206	22 (10.7)	15 (8.9)	7 (18.9)
Pharmacist/assistant pharmacist	206	19 (9.2)	13 (7.8)	6 (15.0)
Childcare worker	185	12 (6.5)	7 (5.0)	5 (11.4)
Journalist	179	5 (2.8)	4 (2.5)	1 (5.9)
Kitchen staff	159	24 (15.1)	23 (16.1)	1 (6.3)
Other function	159	11 (6.9)	8 (5.4)	3 (30.0)
Cashier	139	11 (7.9)	5 (4.4)	6 (23.1)
Domestic care worker	130	7 (5.4)	3 (2.7)	4 (21.1)
Farmer/gardener	104	15 (14.4)	9 (9.9)	6 (46.2)
Communication/marketing manager	103	13 (12.6)	11 (12.5)	2 (13.3)
Bank/post office teller	100	10 (10.0)	7 (8.0)	3 (25.0)
Communication marketing manager	99	10 (10.1)	8 (9.2)	2 (16.7)
Home delivery driver	42	4 (9.5)	3 (8.8)	1 (12.5)
Taxi driver/chauffeur	36	4 (11.1)	3 (8.8)	1 (50.0)
Teacher	35	3 (8.6)	2 (6.9)	1 (16.7)
Undertaker	33	5 (15.2)	5 (15.2)	-

Supplementary table 6. Sample description and prevalence of anti-SARS-CoV-2 IgG antibodies, including relative risks further adjusted for out-of-work exposure to confirmed COVID-19 cases, SEROCoV-WORK+ study, May-September 2020, Geneva, Switzerland

	N (%)	Seropositive	Relative Risk (95% CrI)
Total	10494	1023 (9.8)	---
Women	5840 (55.7)	596 (10.2)	1.00 (ref.)
Men	4654 (44.3)	427 (9.2)	0.93 (0.80-1.07)
Age group, years			
18-34	2675 (25.5)	291 (10.9)	1.00 (ref.)
35-49	4520 (43.1)	466 (10.3)	0.98 (0.83-1.16)
50-65	3299 (31.4)	266 (8.1)	0.73 (0.60-0.88)
Educational level[a]			
Mandatory education	865 (8.2)	87 (10.1)	0.75 (0.51-1.06)
Apprenticeship	2215 (21.1)	175 (7.9)	0.52 (0.35-0.73)
Secondary education	2096 (20.0)	194 (9.3)	0.65 (0.46-0.89)
University	4619 (44.0)	490 (10.6)	0.75 (0.55-1.00)
Doctorate	413 (3.9)	52 (12.6)	1.00 (ref.)
Other	282 (2.7)	25 (8.9)	---
Activity sector[b]			
Healthcare	1668 (15.9)	185 (11.1)	1.00 (ref.)
Transportation	1184 (11.3)	99 (8.4)	0.72 (0.40-1.23)
Nursing homes	1098 (10.5)	155 (14.1)	1.21 (0.69-1.92)
Public administration	1055 (10.1)	94 (8.9)	0.87 (0.44-1.56)
Public security	1053 (10.0)	82 (7.8)	0.57 (0.31-0.94)
Food industry	754 (7.2)	76 (10.1)	0.95 (0.49-1.66)
Homecare	753 (7.2)	91 (12.1)	0.97 (0.36-2.00)
Social work	734 (7.0)	62 (8.4)	0.72 (0.34-1.36)
Financial services	528 (5.0)	65 (12.3)	1.05 (0.48-1.91)
International organizations	419 (4.0)	24 (5.7)	0.49 (0.14-1.42)
Early childhood education	259 (2.5)	15 (5.8)	0.39 (0.13-0.85)
Pharmacy	254 (2.4)	28 (11.0)	0.85 (0.40-1.52)
Construction	181 (1.7)	11 (6.1)	0.59 (0.13-1.60)
Media	166 (1.6)	7 (4.2)	0.60 (0.09-2.09)
Agriculture	97 (0.9)	8 (8.2)	0.77 (0.22-1.84)
Other	291 (2.8)	21 (7.2)	0.57 (0.22-1.10)
Confinement status[c]			
Mobilized/partially confined	9418 (89.8)	898 (9.5)	1.00 (ref.)
Fully confined	1065 (10.2)	125 (11.7)	1.28 (1.04-1.56)
Out-of-work exposure[d]			
0	9129 (87.0)	775 (8.5)	1.00 (ref.)
≥1	1365 (13.0)	248 (18.2)	2.29 (1.93-2.74)
Smoking			
Non-smoker	6116 (58.3)	708 (11.6)	1.00 (ref.)
Ex-smoker	1662 (16.4)	143 (8.6)	0.71 (0.55-0.86)
Smoker	2714 (23.1)	172 (6.3)	0.45 (0.33-0.56)
BMI group			
18-24.9	5742 (56.1)	584 (10.2)	1.00 (ref.)
25-29.9	3271 (23.8)	305 (9.3)	0.96 (0.82-1.13)
≥30	1220 (10.6)	114 (9.3)	0.94 (0.73-1.15)
Chronic conditions[e]			
None	9267 (88.3)	900 (9.7)	1.00 (ref.)
1	1060 (10.0)	106 (10.0)	1.10 (0.85-1.38)
≥2	166 (1.6)	17 (10.2)	1.23 (0.67-1.94)

Results are N (%), or as stated. Relative risks (95% credible interval) are from Bayesian logistic regression models, and are adjusted for test performance, age, sex and out-of-work exposure to confirmed COVID-19 cases. These pertain to the reference individual (Female, 18-34 years, no out-of-work exposure). 69 participants were excluded due to missing serology or sociodemographic data, or due to being outside target age range of 18-65 years. All data are self-reported except serology.
[a] Each category indicates the highest level of education attained by participant; mandatory education indicates 15 years of primary education as the highest obtained degree.
[b] See Supplementary Table 1 for details.

[c] Out-of-work exposure to confirmed COVID-19 cases.
[d] Chronic conditions: hypertension, diabetes, cardiovascular disease, respiratory disease, cancer.

Supplementary table 7. Seropositivity rate according to testing site, SEROCoV-WORK+ study, May-September 2020, Geneva, Switzerland

Testing site	N	Seropositive n (%)
Testing site 1	3065	280 (9.1)
Testing site 2	2186	235 (10.8)
Testing site 3	3004	276 (9.2)
On-site testing	2258	235 (10.4)

Supplementary figure 1. Prevalence of anti-SARS-CoV-2 IgG antibodies by sector excluding participants who were fully confined during the lockdown, SEROCoV-WORK+ study, May-September 2020, Geneva, Switzerland (N = 9430)

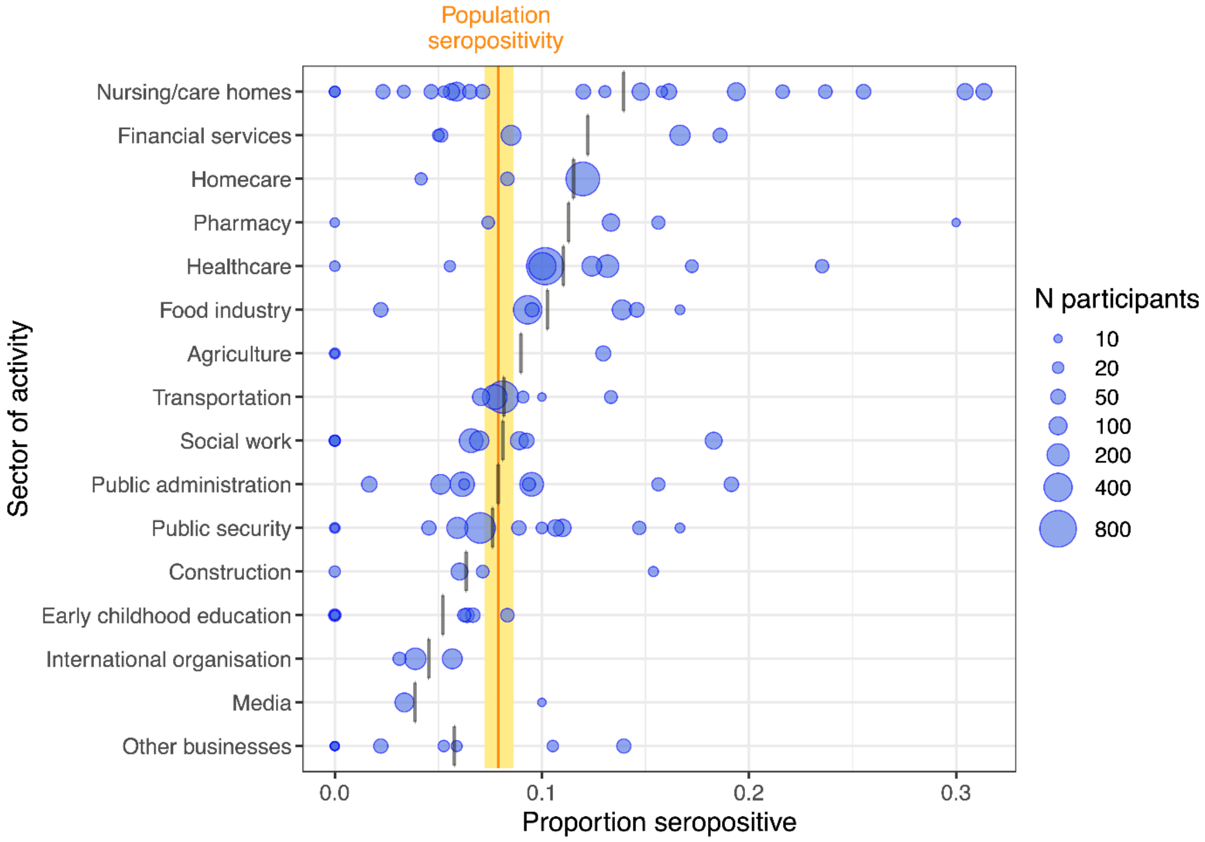

Sample size: 9430 participants, 899 of which were seropositive.
Blue dots represent proportion of seropositive participants per company/workplace setting.
Dot size indicates number of employees participating.
Darker dots indicate more than one facility with same or very similar seropositivity rate.
Vertical orange bar and yellow area indicate general working-age population seropositivity rate and 95% binomial confidence interval, respectively, from SEROCoV-POP study (20,21).
Small grey vertical bars show the proportion positive of all participants per sector.
Facilities with < 10 participants are not shown as dots, but these participants are included in the sector average.

Supplementary figure 2. Prevalence of anti-SARS-CoV-2 IgG antibodies by occupation, separately for all participants (N = 10,513) versus only mobilized participants (N = 9430), SEROCoV-WORK+ study, May-September 2020, Geneva, Switzerland

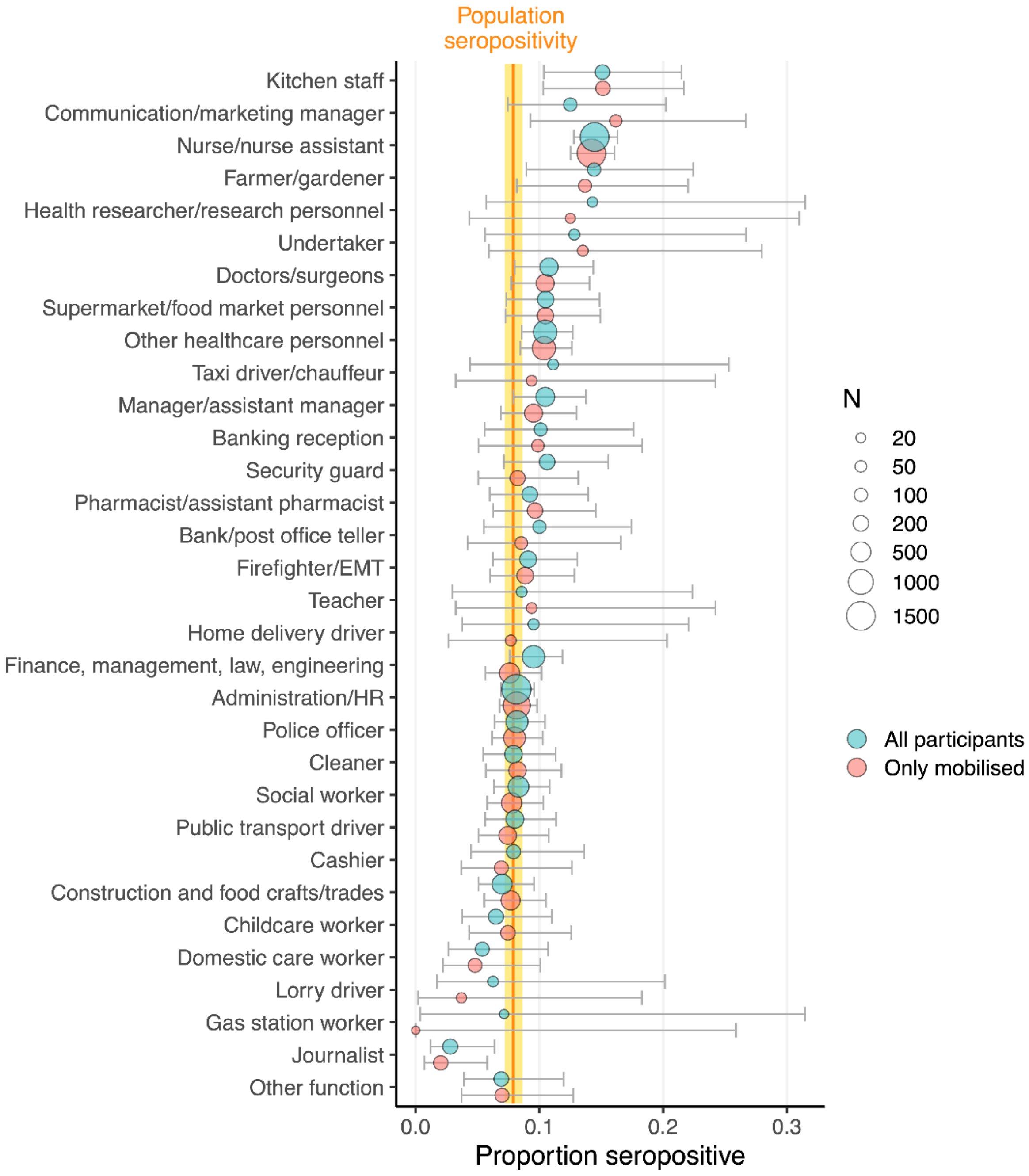

Sample size: 10513 participants, 1026 of which were seropositive.
Dots indicate mean seropositivity rate for each occupation group, while horizontal grey lines represent 95% binomial confidence intervals. Green dot indicates all participants; orange dot indicates only mobilized participants (excluding participants who were fully confined during the lockdown).
Dot size indicates number of employees with that occupation.
Vertical orange bar and yellow area indicate general working-age population seropositivity rate and 95% binomial confidence interval, respectively, from SEROCoV-POP study (20,21).

Supplementary figure 3. Weekly number of virologically-confirmed SARS-CoV-2 infections in the canton of Geneva between March and September, 2020, and weekly number of participants tested in the SEROCoV-WORK+ study between May 18 and September 18, 2020

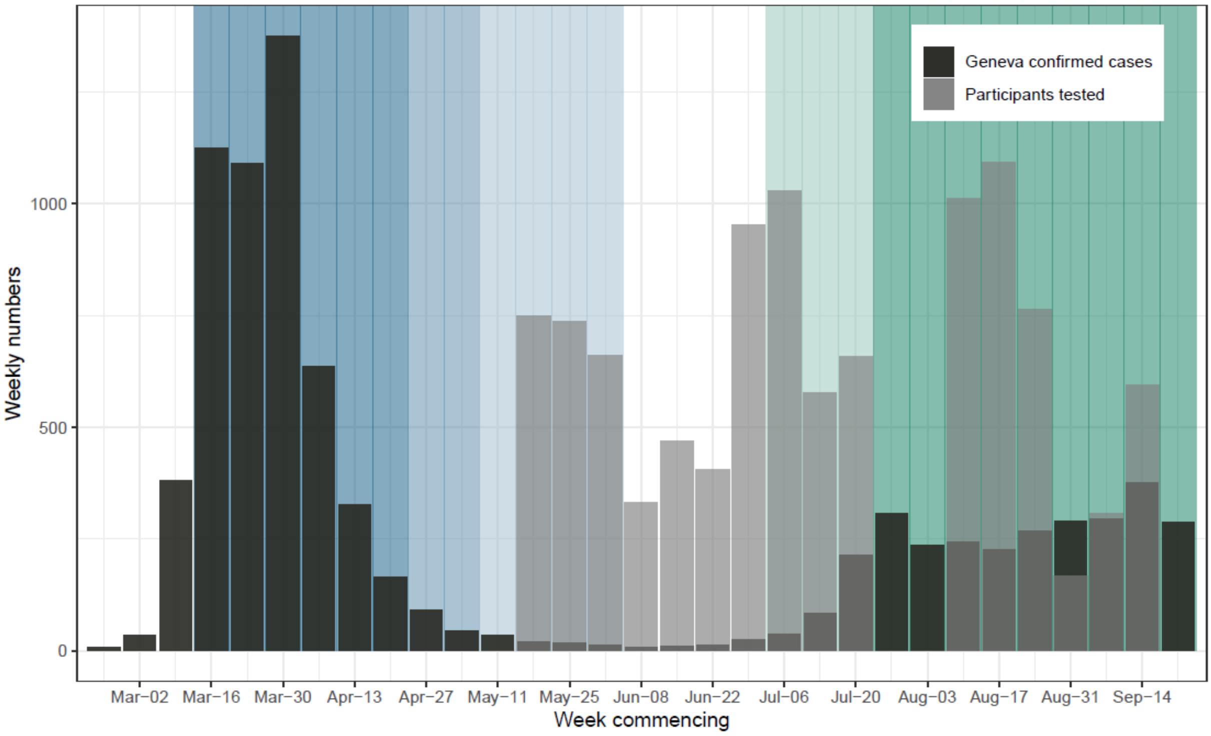

Background shading indicates government-mandated prevention measures in place:

Darker blue	Lockdown imposed in canton Geneva on March 16, 2020.
Middle blue	First phase of easing lockdown measures began on April 27, 2020.
Lighter blue	Second phase of easing lockdown measures on began on May 11, 2020.
No shade	All lockdown measures lifted in canton Geneva on June 6, 2020.
Lighter green	Federally-mandated mask wearing in public transport began on July 6, 2020 (ongoing as of March 10, 2021).
Darker green	Mask wearing inside stores became mandatory in canton Geneva on July 28, 2020 (ongoing as of March 10, 2021).

Virologically-confirmed SARS-CoV-2 infection data from the Canton of Geneva:
https://infocovid.smc.unige.ch/

Supplementary figure 4. Prevalence of anti-SARS-CoV-2 IgG antibodies by activity sector, SEROCoV-WORK+ study, May-September 2020, Geneva, Switzerland

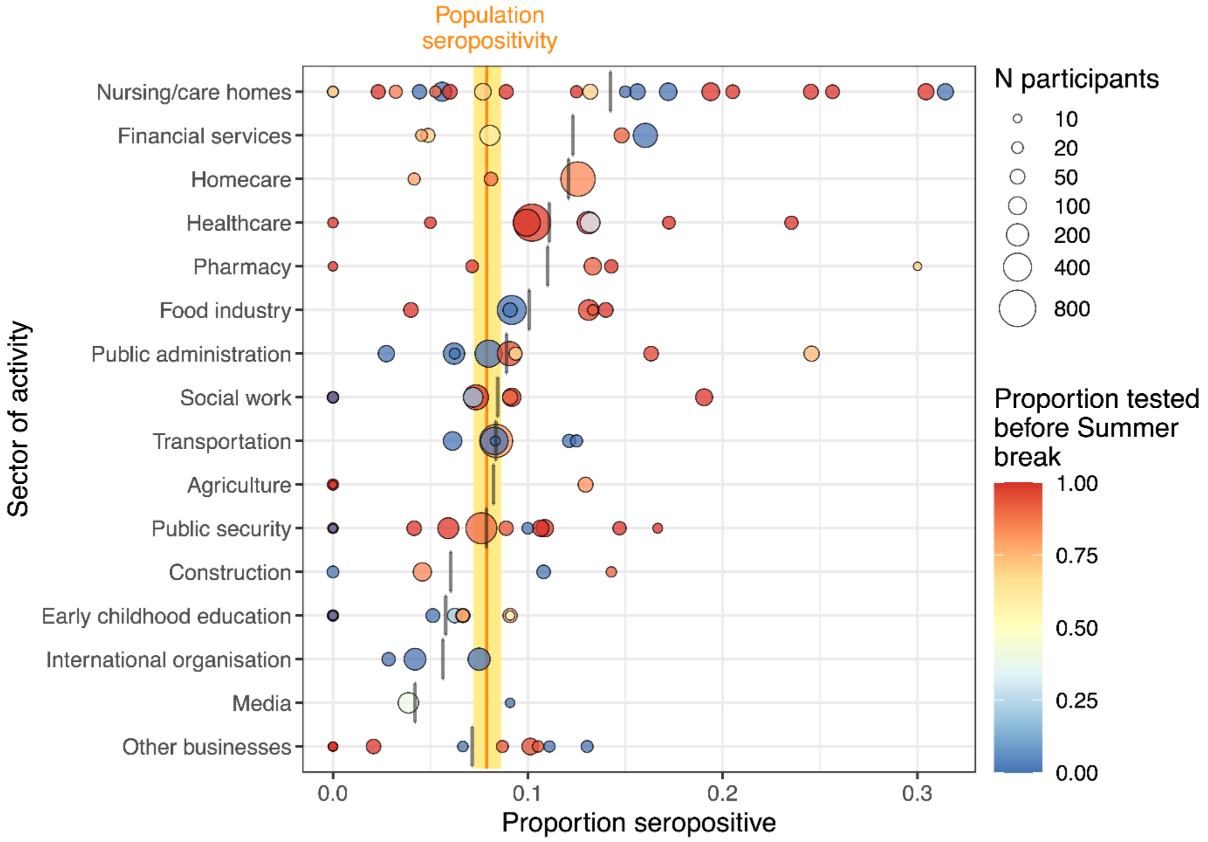

Sample size: 10513 participants, 1026 of which were seropositive.
Dots represent proportion of seropositive participants per company/workplace facility.
Dot size indicates number of employees participating.
Dot color indicates the proportion of participants at each facility who were tested before summer break.
Vertical orange bar and yellow area indicate general working-age population seropositivity rate and 95% binomial confidence interval, respectively, from SEROCoV-POP study (20,21).
Small grey vertical bars show the proportion positive of all participants per sector.
Facilities with < 10 participants are not shown as dots, but these participants are included in the sector average.

Supplementary figure 5. Posterior distributions of sector-specific mean estimates

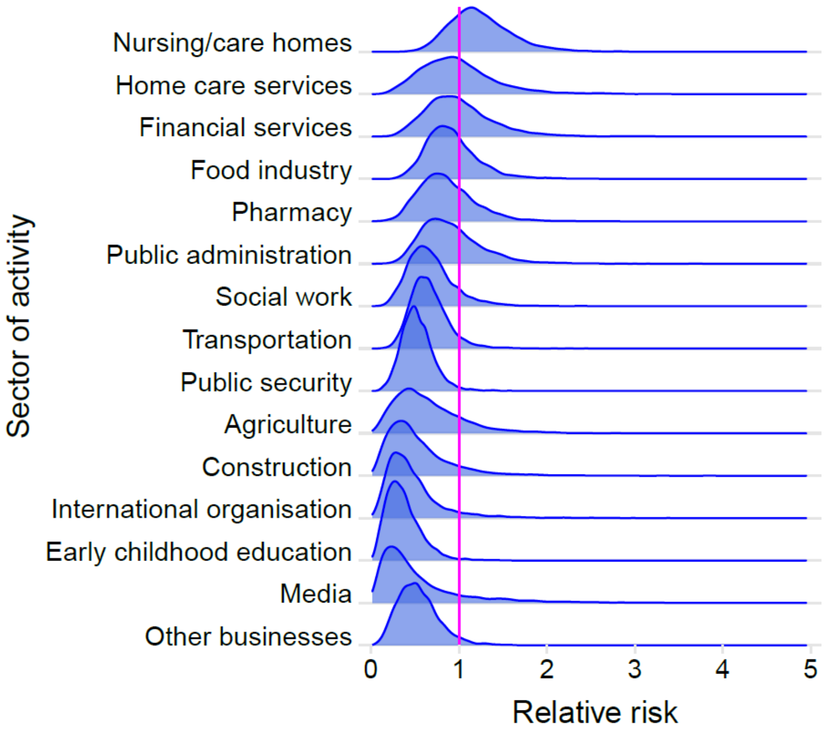

Magenta line is the reference healthcare sector.

Supplementary figure 6. Posterior distributions of sector-specific variance estimates

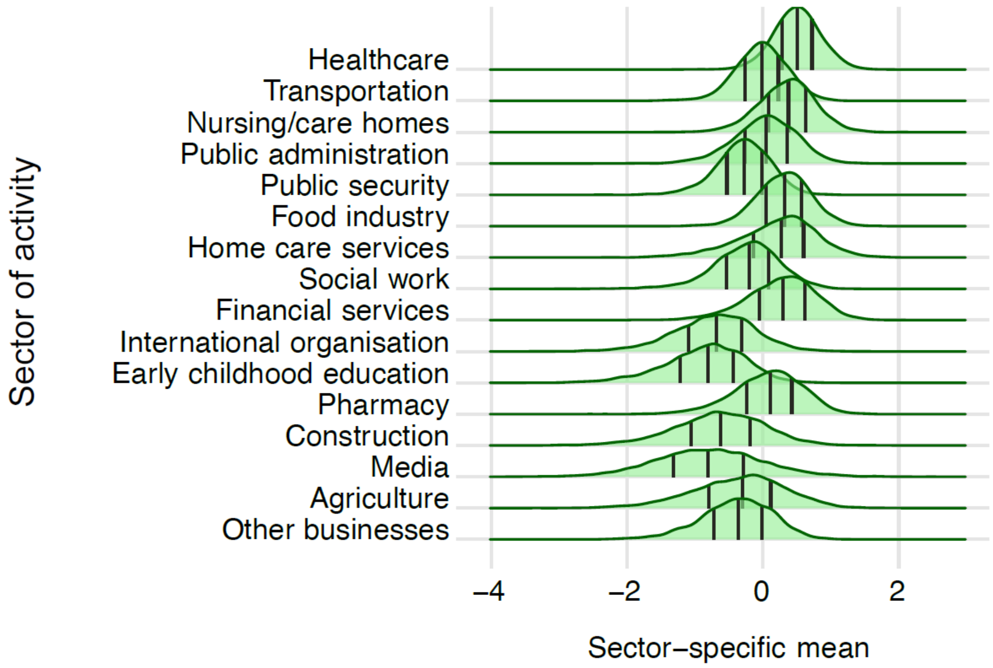

Black vertical lines in each sector indicate the quartiles of the estimated densities.

Supplementary figure 7. Posterior parameter estimates of sector-specific variance

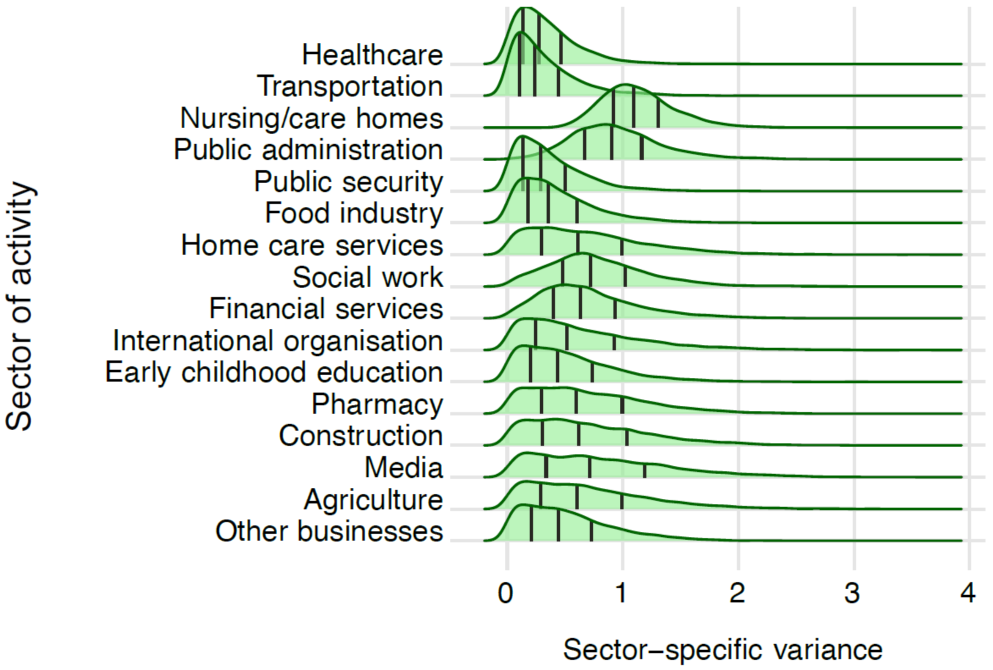

Black vertical lines in each sector indicate the quartiles of the estimated densities.

Supplementary References

1 Stringhini S, Wisniak A, Piumati G *et al*. Seroprevalence of anti-SARS-CoV-2 IgG antibodies in Geneva, Switzerland (SEROCoV-POP): a population-based study. *The Lancet* 2020; **396**: 313-9.

2 Meyer B, Torriani G, Yerly S *et al*. Validation of a commercially available SARS-CoV-2 serological immunoassay. *Clin Microbiol Infect*. 2020; **26**(**10**):1386-1394.
