# Supplementary material for: Large variation in anti-SARS-CoV-2 antibody prevalence among essential workers in Geneva, Switzerland
Source: Nat Commun. 2021 Jun 8;12:3455. doi: 10.1038/s41467-021-23796-4 (PMC8187639; doi:10.1038/s41467-021-23796-4)
Supplement: Supplementary file 3 — Reporting Summary [file 41467_2021_23796_MOESM3_ESM.pdf]

nature research

Corresponding author(s): Silvia Stringhini

Last updated by author(s): Mar 11, 2021

Reporting Summary

Nature Research wishes to improve the reproducibility of the work that we publish. This form provides structure for consistency and transparency in reporting. For further information on Nature Research policies, see our Editorial Policies and the Editorial Policy Checklist.

Statistics

For all statistical analyses, confirm that the following items are present in the figure legend, table legend, main text, or Methods section.

n/a	Confirmed	
☐	☒	The exact sample size (*n*) for each experimental group/condition, given as a discrete number and unit of measurement
☐	☒	A statement on whether measurements were taken from distinct samples or whether the same sample was measured repeatedly
☐	☒	The statistical test(s) used AND whether they are one- or two-sided<br>*Only common tests should be described solely by name; describe more complex techniques in the Methods section.*
☐	☒	A description of all covariates tested
☐	☒	A description of any assumptions or corrections, such as tests of normality and adjustment for multiple comparisons
☐	☒	A full description of the statistical parameters including central tendency (e.g. means) or other basic estimates (e.g. regression coefficient) AND variation (e.g. standard deviation) or associated estimates of uncertainty (e.g. confidence intervals)
☐	☒	For null hypothesis testing, the test statistic (e.g. *F*, *t*, *r*) with confidence intervals, effect sizes, degrees of freedom and *P* value noted<br>*Give P values as exact values whenever suitable.*
☐	☒	For Bayesian analysis, information on the choice of priors and Markov chain Monte Carlo settings
☒	☐	For hierarchical and complex designs, identification of the appropriate level for tests and full reporting of outcomes
☒	☐	Estimates of effect sizes (e.g. Cohen's *d*, Pearson's *r*), indicating how they were calculated

Our web collection on statistics for biologists contains articles on many of the points above.

Software and code

Policy information about availability of computer code

Data collection	REDCap sofwatre v9.1.0
Data analysis	R version 4.0.3; packages: rstan (version 2.21.2) and shinystan (version 2.5.0)

For manuscripts utilizing custom algorithms or software that are central to the research but not yet described in published literature, software must be made available to editors and reviewers. We strongly encourage code deposition in a community repository (e.g. GitHub). See the Nature Research guidelines for submitting code & software for further information.

Data

Policy information about availability of data

All manuscripts must include a data availability statement. This statement should provide the following information, where applicable:
- Accession codes, unique identifiers, or web links for publicly available datasets
- A list of figures that have associated raw data
- A description of any restrictions on data availability

Due to cantonal regulations, our data are not immediately available. However, our data can be made available to share upon submission of a data request application to the investigators board via the corresponding author. Virologically-confirmed SARS-CoV-2 infection data from the Canton of Geneva: https://www.ge.ch/document/covid-19-situation-epidemiologique-geneve.

Field-specific reporting

Please select the one below that is the best fit for your research. If you are not sure, read the appropriate sections before making your selection.

☒ Life sciences ☐ Behavioural & social sciences ☐ Ecological, evolutionary & environmental sciences

For a reference copy of the document with all sections, see nature.com/documents/nr-reporting-summary-flat.pdf

Life sciences study design

All studies must disclose on these points even when the disclosure is negative.

Sample size	No sample-size calculation was done. All workers that were mobilized during the lockdown and who worked in the companies/institutions included in a list we created were invited to participate.
Data exclusions	No data were excluded.
Replication	Due to the large amount of samples to be processed, all testing using the Euroimmun ELISA were performed only once in the study. Accordingly, relative risk analyses were adjusted for test performance, as described in the methods.
Randomization	This is an observational study without an intervention, so randomization is not applicable.
Blinding	This is an observational study without an intervention, so blinding is not applicable.

Reporting for specific materials, systems and methods

We require information from authors about some types of materials, experimental systems and methods used in many studies. Here, indicate whether each material, system or method listed is relevant to your study. If you are not sure if a list item applies to your research, read the appropriate section before selecting a response.

Materials & experimental systems

n/a	Involved in the study	
☐	☒	Antibodies
☒	☐	Eukaryotic cell lines
☒	☐	Palaeontology and archaeology
☒	☐	Animals and other organisms
☐	☒	Human research participants
☒	☐	Clinical data
☒	☐	Dual use research of concern

Methods

n/a	Involved in the study	
☒	☐	ChIP-seq
☒	☐	Flow cytometry
☒	☐	MRI-based neuroimaging

Antibodies

Antibodies used	We assessed anti-SARS-CoV-2 IgG antibodies using a commercially available ELISA (Euroimmun; Lübeck, Germany #EI 2606-9601 G) targeting the S1 domain of the virus spike protein, using the manufacturer’s recommended cut-off of ≥1.1 for seropositivity
Validation	Meyer, B. et al. Validation of a commercially available SARS-CoV-2 serological immunoassay. Clin. Microbiol. Infect. 26, 1386–1394 (2020).

Human research participants

Policy information about studies involving human research participants

Population characteristics	Working-age adults who were mobilized during the Spring 2020 lockdown in Switzerland: 10,513 participants, of which 55.6% women, and with a mean age [standard deviation]: 43.0 years [10.7].
Recruitment	We selected public and private companies and institutions that were potentially mobilized (see Supplementary Table 1 for further selection and recruitment information). Participating facilities in turn invited their employees to participate on a voluntary basis. All participants gave written informed consent, completed a questionnaire and provided a venous blood sample. While the sample was not randomly selected from the general population, the overall seropositivity rate reflected that of the general population. Participation rate was around 50% for most sectors, also reflecting participation rates in population-based surveys. Thus, self-selection and participation bias may be present but likely no differently than in popultion-based representative samples.
Ethics oversight	The Cantonal Research Ethics Commission of Geneva, Switzerland, approved the study protocol (project number 2020-00881).

Note that full information on the approval of the study protocol must also be provided in the manuscript.
